# Atlas of Skin Diseases

**Published:** 1876-09

**Authors:** 


Atlas of Skin Diseases. By Louis A. Duhring, M.D., Professor of Skin Diseases in the Hospital of the University of Pennsylvania, Physician to the Dispensary for Skin Diseases, Philadelphia, etc. Price $2.50 per part. J. B. Lippincott & Co., publishers, 715 and 717 Market Street, Philadelphia.

We are in receipt of part first, of a series of eight or ten parts to follow, of the above publication, and from careful scrutiny of its contents, we feel assured in saying that it will be of great value to the general practitioner, and to those who have not extensive clinical advantages for studying diseases of the skin. The importance of a thorough knowledge of this class of diseases has been greatly unappreciated by the profession; but it is now being realized that skin diseases demand a share of our studies, and many are making this branch a specialty.

Dr. Duhring, in his announcement, calls attention to the fact, as he claims, that the same diseases of the skin in this country and in Europe are quite dissimilar in their appearance. Each part will contain a number of typical representations of various diseases. The photographs will be nearly life size, and taken from nature, therefore will be an exact likeness, which, combined with a clear and concise description of the individual diseases, giving cause, history and treatment, renders his atlas almost, or quite, as instructive and impressive as if the patient were before you.

The atlas will be issued in parts, quarterly; each part containing four plates. The first part contains, Eczema (Erythematomosum), Psoriasis, Lupus Erythematomosum, and Syphiloderma Pustalosum, diseases with which any physician will be likely to meet any day, and he should be prepared to diagnose and treat them successfully.

